# Research progress on *Avibacterium paragallinarum* and related bacterial and viral diseases in poultry and their mixed infections

**DOI:** 10.3389/fmicb.2026.1857529

**Published:** 2026-06-18

**Authors:** Chen Mei, Mingjiang Liu, Xingmin Pan, Ying Liu, Zhaoling Jiang, Zhihua Li, Zhenyi Liu, Baozhu Xing, Shijie Xie, Xueze Lyu, Hongjun Wang

**Affiliations:** 1 Institute of Animal Husbandry and Veterinary Medicine, Beijing Academy of Agriculture and Forestry Sciences, Beijing, China; 2 College of Veterinary Medicine, Jiangsu Co-Innovation Center for Prevention and Control of Important Animal Infectious Diseases and Zoonoses, Yangzhou University, Yangzhou, China; 3 Wuhan Institute of Virology, Chinese Academy of Sciences, Wuhan, China; 4 Beijing General Animal Husbandry Station, Beijing, China

**Keywords:** antigen, *Avibacterium paragallinarum*, bacterial and viral diseases, drug control, mixed infections, vaccine development

## Abstract

*Avibacterium paragallinarum* (*A. paragallinarum*) is one of the primary pathogens causing respiratory diseases in poultry flocks, posing a serious threat to the health and production efficiency of the global poultry industry. With the expansion of modern farming scales and changes in environmental factors, mixed infections involving *A. paragallinarum* alongside other bacteria and viruses have become increasingly prevalent. This has led to more complex and diverse disease manifestations, significantly increasing the difficulty of prevsention and control. Current research on *A. paragallinarum* encompasses its antigenic structural characteristics, vaccine development and optimization, and therapeutic strategies. However, gaps remain in the profound understanding of mixed infection mechanisms and synergistic control approaches. This manuscript systematically summarizes recent advances in research concerning *A. paragallinarum* and its associated bacterial and viral co-infections. It focuses on analyzing the impact of antigenic variation on vaccine efficacy, the status of composite vaccine development, and the pathological mechanisms of multi-pathogen synergistic infection. The aim is to provide a theoretical foundation and practical reference for the comprehensive prevention and control of *A. paragallinarum*-related diseases, thereby promoting the healthy and sustainable development of the poultry farming industry.

## Graphical abstract

**Figure:**
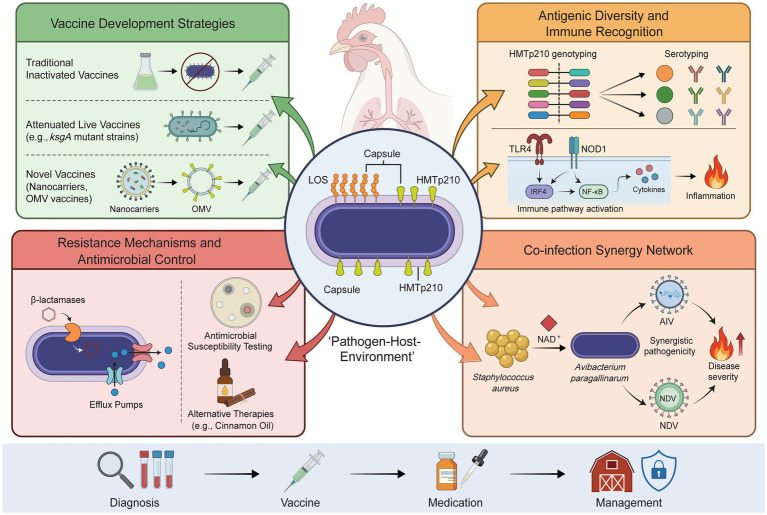
Integrated mechanism diagram of *Avibacterium paragallinarum* infection and control.

## 1 Background

*Avibacterium paragallinarum* is the primary pathogen responsible for infectious coryza (IC) in chickens. This acute, highly contagious respiratory disease inflicts substantial economic losses upon the poultry industry (Shelkamy et al., 2025a). In recent years, with the expansion of farming scales and the increasing complexity of rearing environments, cases of *Avibacterium paragallinarum* infection have increased. Concurrently, mixed infections with other bacteria and viruses have emerged, rendering the clinical manifestations of the disease more complex and significantly increasing the difficulty of diagnosis and treatment (Mei et al., 2020a,b).

*A. paragallinarum* exhibits considerable genetic and biochemical diversity, with strains isolated from different geographic regions displaying variations in genetic characteristics, antigenicity, and pathogenicity. For example, strains of *A. paragallinarum* isolated within China demonstrate differences from international reference strains in both 16S rRNA sequences and HMTp210 gene sequences, alongside distinct biochemical properties (Chen et al., 2025). This diversity poses challenges for vaccine development and disease control, particularly concerning the limited cross-protective capacity of vaccines (Caballero-Garcia et al., 2022). Furthermore, the pathogenicity of *A. paragallinarum* is influenced by strain variation, with different strains exhibiting distinct capacities to induce clinical symptoms, replicate, and cause mortality (Mei et al., 2023).

In terms of diagnosis, traditional bacterial culture methods face limitations due to the slow growth of *A. paragallinarum* and its requirement for specific nutritional conditions, resulting in prolonged diagnostic cycles and limited success. To enhance diagnostic efficiency and specificity, molecular biological techniques such as real-time quantitative PCR (qPCR) have gained widespread application in recent years. However, recent studies have identified non-pathogenic *A. paragallinarum* strains capable of colonizing asymptomatic poultry populations, potentially leading to false-positive results in routine qPCR assays. Consequently, researchers have developed specific PCR methods to differentiate pathogenic from non-pathogenic strains; nevertheless, complete differentiation remains challenging due to strain diversity (Shelkamy et al., 2025b). Furthermore, to meet the demand for rapid on-site detection, nucleic acid hybridization lateral flow assays have been established for the rapid and sensitive detection of *A. paragallinarum* nucleic acids (Huo et al., 2021a,b). This technology exhibits good sensitivity and specificity, making it suitable for early clinical diagnosis and field screening (Kuchipudi et al., 2021).

Pathologically, *A. paragallinarum* primarily invades the upper respiratory tract of chickens, causing inflammation of the nasal cavity and wing sinuses, accompanied by copious mucus production and secretion. Research indicates that following infection, the host innate immune response is significantly activated, particularly through upregulation of the TLR4 and NOD1 signaling pathways. Pro-inflammatory cytokines such as IL-1β and IL-6 are extensively expressed in local tissues, driving acute inflammatory responses. Elevated levels of pro-inflammatory cytokines in the nasal cavity during early infection are considered a key factor in acute upper respiratory tract inflammation (Guo et al., 2022a,b). Concurrently, infection may induce systemic immune cell dysfunction, such as monocytosis with downregulated surface MHC-II molecule expression and impaired lymphocyte function, potentially increasing susceptibility to secondary infections and exacerbating clinical symptoms (Alvarez et al., 2020).

Moreover, interactions between *A. paragallinarum* and host commensal bacteria (such as *Staphylococcus* species) promote its survival and dissemination, revealing the critical role of the resident microbiota in opportunistic respiratory infections. Specifically, *Staphylococcus chromogenes* significantly enhances the infectivity of *A. paragallinarum*. This bacterium not only directly supplies the essential growth factor nicotinamide adenine dinucleotide (NAD^+^), but also accelerates the synthesis and release of NAD^+^ within host cells, thereby promoting the survival and proliferation of *A. paragallinarum*. Targeted antibiotic intervention against *S. chromogenes* effectively disrupts the infection process, suggesting that modulating resident bacterial dynamics may represent a novel strategy for preventing and controlling opportunistic infections. The study further elucidated how opportunistic pathogens exploit resident bacteria to facilitate infection and spread in hosts with compromised immune systems or disrupted normal microbiota. Observations in avian models revealed that *S. chromogenes* plays a pivotal role in promoting *A. paragallinarum* infection, identifying complex interactions between resident bacteria and opportunistic pathogens. This discovery offers novel insights into the mechanisms of respiratory infections, as well as providing a theoretical foundation for developing novel therapeutic strategies (Wu et al., 2021).

**Figure 1:**
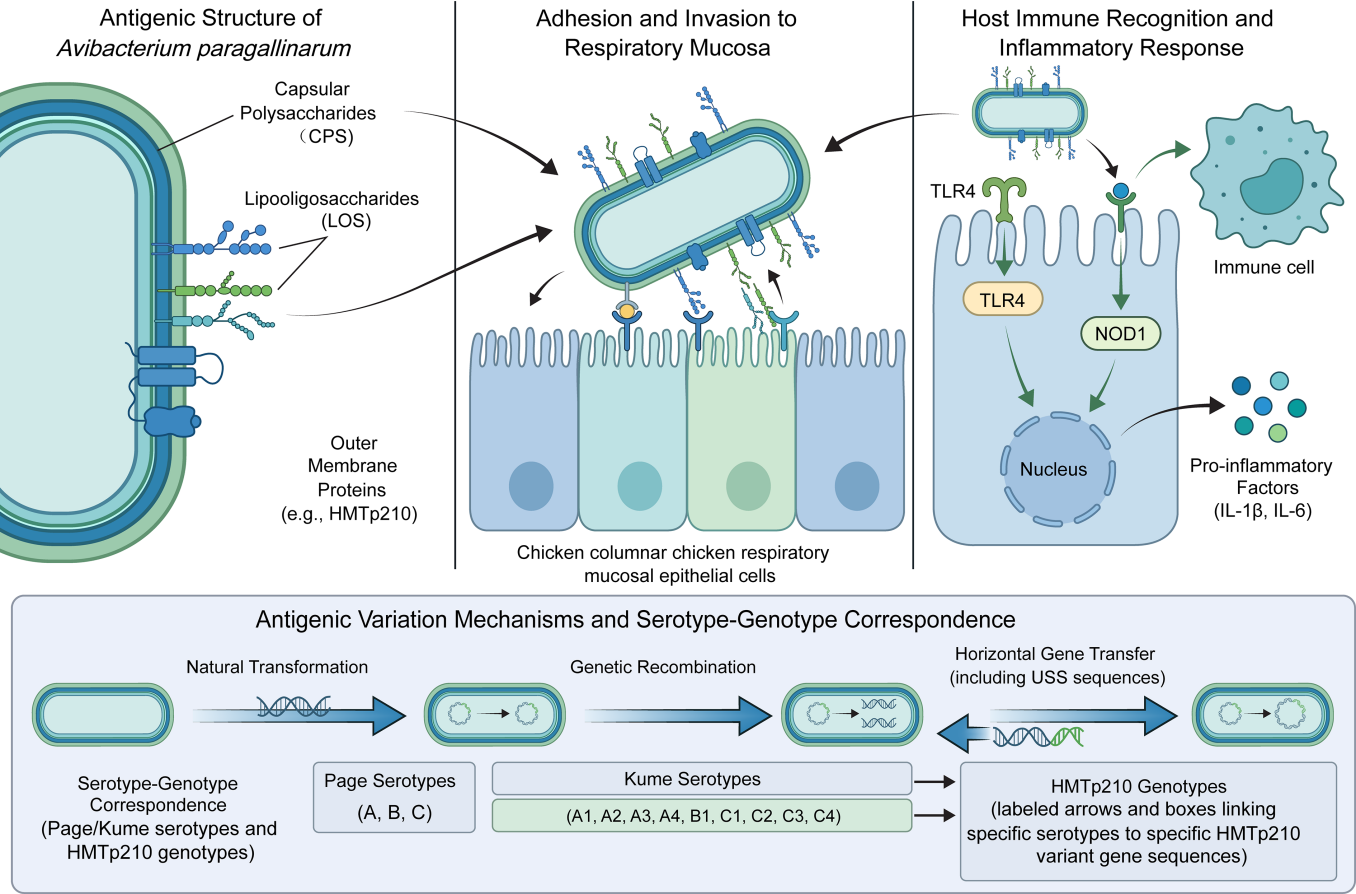
Antigenic structure and immunological recognition of *A. paragallinarum.*

The pathogenicity of *A. paragallinarum* is closely associated with its biofilm formation capacity, and lipopolysaccharide and capsular polysaccharide structures (Chiang et al., 2013). Deletion of relevant genes significantly impacts biofilm formation and antiserum killing capacity, thereby reducing pathogenicity. Chen et al. identified and characterized gene clusters associated with lipooligosaccharide and capsular polysaccharide synthesis in *A. paragallinarum*. The study revealed that gene cluster L6, along with the *waaF* and *waaQ* genes, are involved in LOS synthesis, while the *acbD* and *ccbF1* genes participate in capsular polysaccharide production. Mutant and compensatory strains were generated via natural transformation. Mutant strains lacking these genes exhibit significantly lower survival rates in chicken serum compared to wild-type strains, indicating that those genes play crucial roles in *A. paragallinarum* pathogenicity and immune evasion (Zhao et al., 2001) (see Figure 1).

Further studies revealed that mutant strains lacking the *waaF*, *waaQ*, *L6*, *acbD*, and *ccbF1* genes exhibited significantly enhanced biofilm formation without affecting immunogenicity in chickens. Those mutants demonstrate increased susceptibility to the antimicrobial peptide fowlicidin-2 and reduced pathogenicity in chickens. The findings provide crucial foundational data for elucidating the pathogenic mechanisms of *A. paragallinarum,* and establish a technical basis for novel vaccine development (Chen et al., 2024). Huo et al. (2021a,b) used transcriptomic and bioinformatics analyses to investigate the regulatory mechanisms governing iron acquisition and heme utilization in *A. paragallinarum* under iron-deficient conditions. The study identified numerous differentially expressed genes (DEGs) in the iron-deficient group, with most genes downregulated and some expressed exclusively under iron deficient conditions. Analysis indicated that Hut proteins and proteins containing DUF domains are preferentially activated following iron deprivation, playing crucial roles in iron acquisition and heme utilization. These mechanisms provide novel insights for developing vaccine and therapeutic targets.

**Figure 2:**
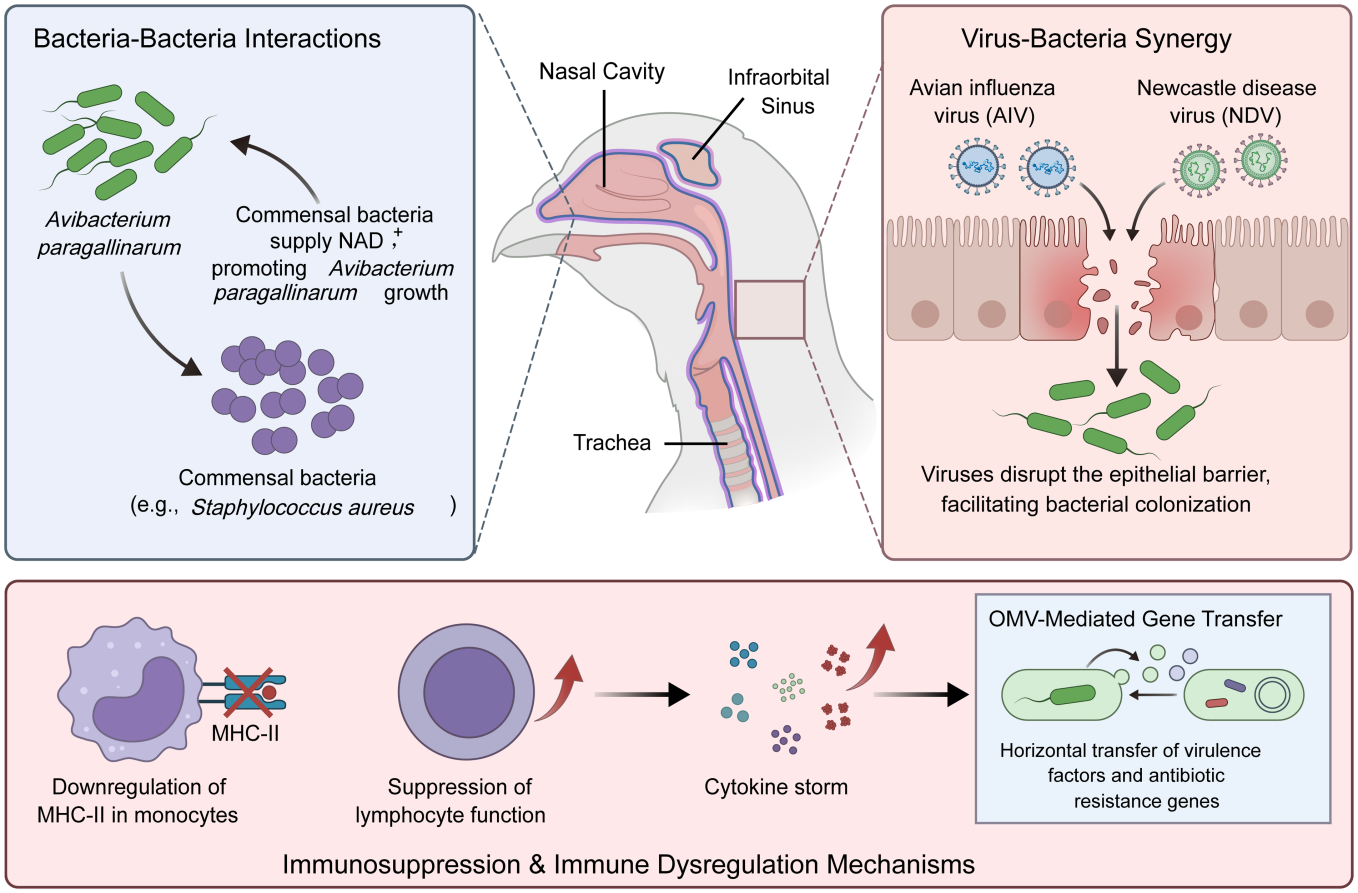
Synergistic pathogenesis mechanism of *A. paragallinarum* mixed infections.

For the prevention and control of *A. paragallinarum*, vaccination currently represents the most effective approach. Previous studies have developed inactivated vaccines by screening strains of varying pathogenicity, significantly reducing clinical symptoms and bacterial colonization (Caballero-Garcia et al., 2022). Concurrently, progress has been made in developing live vaccine candidate strains. Researchers used the Tn5-Kan transposon to construct a transposon mutant library, identifying a mutant strain designated 2019/HB64-40. This strain exhibits disruption of the *ksgA* gene, resulting in reduced biofilm formation capacity, diminished hemagglutinin titers, and restricted growth. Pathogenicity assessments in chickens revealed diminished virulence in the 2019/HB64-40 strain, manifested as milder clinical symptoms and reduced bacterial shedding. Flocks immunized with 2019/HB64-40 demonstrated 90% immune protection following challenge, indicating promising prospects for this attenuated strain as a live vaccine candidate (Guo M. et al., 2025; Guo Y. et al., 2025) (see Figure 2).

**Figure 3:**
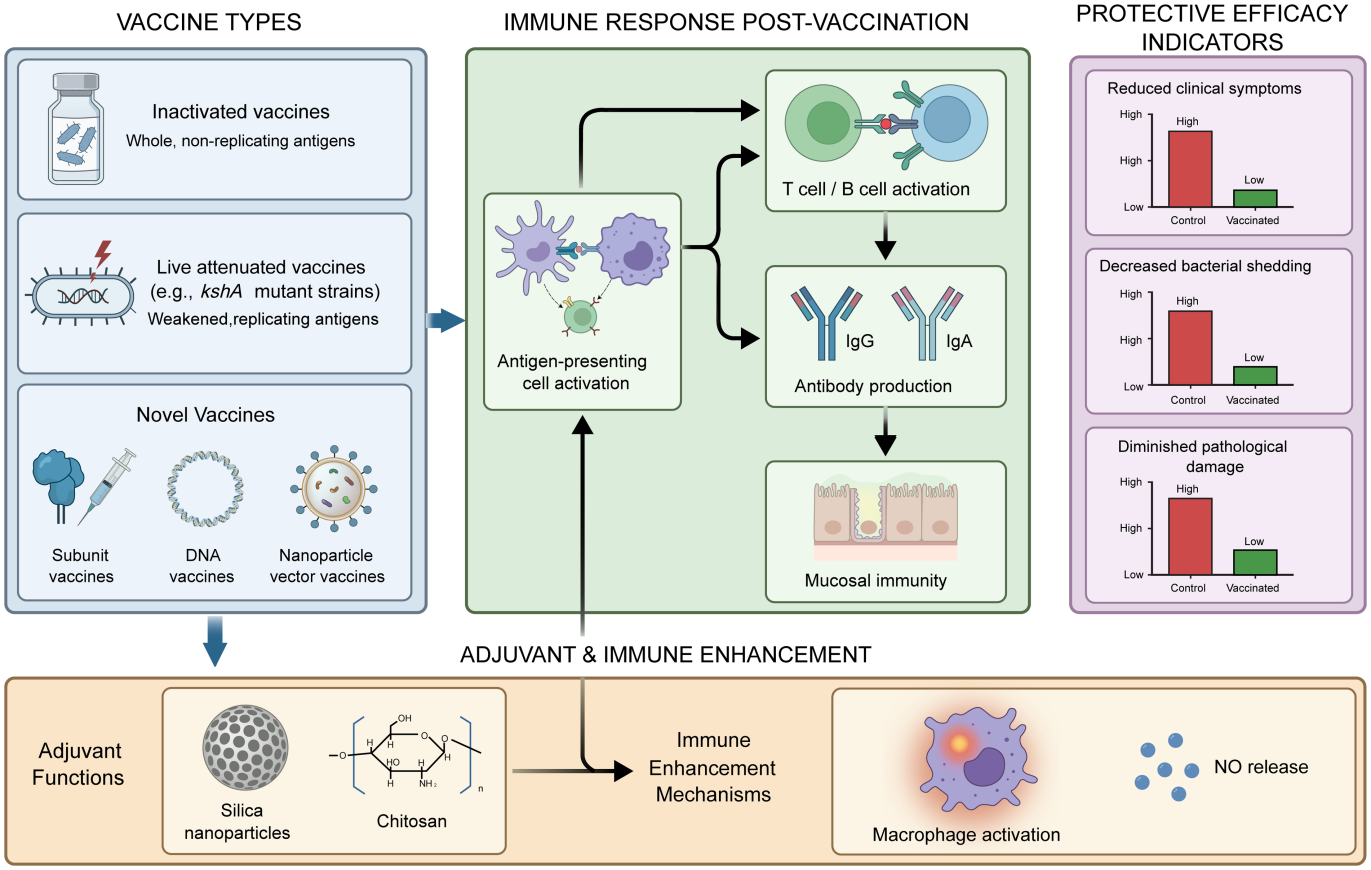
Developmental strategy and immunoprotective mechanism of *A. paragallinarum* vaccines.

Ibrahim et al. investigated the efficacy of various polymeric nanocarriers as adjuvants in enhancing the immunogenicity of vaccines against infectious coryza in chickens. The study revealed that nanoparticles such as silica, iron oxide, and chitosan derivatives at 400 μg/mL exhibited optimal inactivation efficacy against *A. paragallinarum* (Ibrahim et al., 2024). Among those, the adjuvant vaccine incorporating silica nanoparticles elicited the strongest immune response, followed by vaccines containing iron oxide, silica-chitosan composites, and chitosan derivatives. Compared to traditional mineral oil vaccines, nanoparticle adjuvant vaccines demonstrated significant advantages in enhancing immunogenicity. This provides novel insights for developing more effective infectious rhinitis vaccines, particularly in improving immune protection in chickens and controlling disease transmission (Renu and Renukaradhya, 2020) (see Figure 3).

Antimicrobial therapy remains a crucial strategy for controlling *A. paragallinarum* infections, yet the escalating prevalence of resistance renders the establishment of standardized antimicrobial susceptibility testing methods particularly urgent. This study evaluated the impact of different media on antimicrobial susceptibility testing (AST), revealing that the minimum inhibitory concentrations (MIC) obtained after 48 h of incubation in CAMHB + CS + NADH medium exhibited greater consistency, with significantly improved readability and reproducibility compared to 24 h testing. Notably, for 17 antibiotics, the deviation in 48 h MIC values was only ±1-2 dilutions, demonstrating excellent reproducibility. These findings provided a reliable methodological foundation for antibiotic susceptibility testing of *A. paragallinarum*. They also revealed the current prevalence of multiple resistance genes in European and American isolates of that bacterium, providing scientific support for clinical susceptibility testing (Gütgemann et al., 2024).

As the primary pathogen responsible for infectious coryza in chickens, the genetic diversity, pathogenic mechanisms, and mixed infections with other pathogens of *A. paragallinarum* significantly complicate disease prevention and control (Liu et al., 2025). Current research priorities center on developing precise diagnostic techniques, optimizing and innovating vaccines, refining therapeutic strategies, and deepening the understanding of mixed infection mechanisms. Those efforts have considerable significance for enhancing the prevention and control of respiratory diseases in poultry (Etterlin et al., 2023). Through systematic fundamental and applied research, comprehensive prevention and control strategies for *A. paragalliarum*-related diseases can be advanced, providing robust safeguards for the healthy development of the poultry farming industry (Guo M. et al., 2025; Guo Y. et al., 2025).

## 2 Antigenic properties of *A. paragallinarum* and related immunological studies

### 2.1 Antigen structure and classification

*A. paragallinarum*, a key pathogen responsible for infectious coryza in chickens, possesses primary antigenic components, including surface polysaccharides, outer membrane proteins, and multiple virulence factors. These antigenic constituents play a central role in the interaction between the pathogen and the host immune system (Abascal et al., 2009). Surface polysaccharides, as pivotal molecules in the mutual recognition between pathogen and host cells, constitute important targets for inducing specific immune responses.

Outer membrane proteins mediate bacterial adhesion and invasion. Certain specific outer membrane proteins, such as *HMTp210*, have been demonstrated to play a pivotal role in immune responses, making them potential vaccine targets (Li et al., 2023). Furthermore, virulence factors, including secretion system proteins and toxins, not only enhance bacterial pathogenicity, but also serve as candidate targets for immune blockade.

*A. paragallinarum* exhibit significant serotype diversity, which can be classified into multiple serotypes based on serological reactions. Antigenic differences between serotypes are pronounced, posing major challenges for vaccine design and cross-protection. Traditional serotyping methods using Page and Kume serotypes are often limited in practical application due to operational complexity and the representativeness of standard strains (Li et al., 2023). Consequently, gene sequence-based typing methods have gained increasing attention. Research indicates that relying solely on the hypervariable region (HVR) of the *HMTp210* gene cannot distinguish all Page and Kume serotypes. However, combining analyses of gene region 1 with the HVR tandem sequence enables the identification of 14 distinct genotypes (GT ItGT XIV). These genotypes correlate strongly with traditional serotypes and are therefore proposed as a molecular alternative for serotyping. Furthermore, the study indicated that the validity of certain reference strains requires reassessment, particularly for Page serotypes B and C. The findings suggest that tandem sequence analysis of *HMTp210* region 1 and HVR not only enhances the typing capability of *A. paragallinarum* strains, but may also reveal previously unknown Page and Kume serotypes. This approach not only enhances typing resolution, but may also identify novel subtypes not covered by classical serology. It also holds significant application value for epidemiological investigations and diagnostics, while providing more reliable grounds for vaccine antigen selection (Buter et al., 2023). Addressing the antigenic diversity of this bacterium, current research is advancing the development of multivalent vaccines based on outer membrane proteins and surface polysaccharides. This aims to achieve broad protection against different serotypes and enhance the overall efficacy of immunoprophylaxis.

### 2.2 Mechanisms of antigenic variation

*A. paragallinarum* evade host immune surveillance through antigenic variation mechanisms, primarily involving genomic recombination, mutation, and horizontal gene transfer (Xu et al., 2022). Persistent antigenic variation alters bacterial surface antigen structures, rendering existing host immune memory ineffective at recognizing new variants, thereby diminishing the protective efficacy of current vaccines. This mechanism not only complicates infectious disease control, but also indicates that future vaccine development should focus on conserved antigens with broad-spectrum protective potential.

In molecular typing and epidemiological studies, Cao et al. (2024) analyzed the variation characteristics of the *hmtp210* gene through whole-genome sequencing and established a PCR-based typing method for *A. paragallinarum* based on this gene. The study confirmed that the *hmtp210* gene exhibits a high degree of correlation with serotypes of *A. paragallinarum*, enabling effective differentiation between distinct serotypes. This provides crucial evidence for understanding the epidemiological characteristics of antibiotic resistance in *A. paragallinarum* and its transmission patterns within poultry flocks, thereby laying a scientific foundation for the formulation of subsequent control strategies.

One of the primary pathways for antigenic variation is horizontal gene transfer, wherein natural transformation capacity serves as a pivotal component. Liu et al. (2023) demonstrated that *A. paragallinarum* possessed natural transformation capability and established an efficient gene transformation system. Through bioinformatics analysis, the researchers identified 16 capability-associated proteins similar to those found in *A. paragallinarum,* and recognized a substantial number of uptake signal sequences within the genome, ranging between 1,537 and 1,641 in quantity. Experiments demonstrated that plasmids carrying USS exhibited significantly higher transformation efficiency than those without USS, providing a practical tool for subsequent gene functional studies (Wang et al., 2007). The research further revealed that transferring bacteria from nutrient-rich to nutrient-poor media induced their natural transformation capacity. Comparing transformation frequencies across different strains revealed significant variations in efficiency, indicating that selecting appropriate strains was crucial in genetic transformation studies.

### 2.3 Mechanisms of host immune responses induced by *Avibacterium paragallinarum* infection

Infection with *A. paragallinarum* can trigger complex immune responses in the host, involving both cellular and humoral immunity in the defense process, with mucosal immunity being particularly crucial in combating upper respiratory tract infections. Following infection, the host activates multiple immune pathways, inducing an immunological balance between Th1 and Th2 cells, which is essential for effective control of the infection. Research indicates that *A. paragallinarum* infection induces a robust inflammatory response in the upper respiratory tract, accompanied by elevated expression of pro-inflammatory cytokines such as IL-1β and IL-6, particularly in the nasal cavity and paranasal sinuses. This suggests that localized mucosal immune responses constitute a vital barrier against bacterial invasion and dissemination (Guo et al., 2022a,b). The study of Cui et al. (2025) investigated the impact of the probiotic *Enterococcus faecium* (*E. faecium*) on the immunogenicity of vaccines against infectious rhinitis. Results demonstrated that supplementation with *E. faecium* significantly elevated antibody levels in vaccinated chickens and enhanced protective efficacy against serotypes A-1 and C-4 by 6.72 and 7.07%, respectively. Concurrently, *E. faecium* supplementation reduced disease incidence in chickens, indicating its potential application in enhancing vaccine immunogenicity and improving flock health.

## 3 Development of vaccines against *A. paragallinarum*

### 3.1 Traditional inactivated vaccines and live attenuated vaccines

Traditional inactivated vaccines are widely used for the prevention and control of bacterial diseases due to their high safety profiles and excellent stabilities (Xu et al., 2019). These vaccines primarily deliver antigenic material through inactivated whole bacterial cells, stimulating the body to produce a response dominated by humoral immunity. They are suitable for large-scale immunization programs. However, the inactivation process may result in the loss of certain antigenic epitopes, and the duration of immunity is relatively short. Thus, booster doses are typically required to maintain protective efficacy.

To address these limitations, the study focused on optimizing the immunization schedule and evaluating protective efficacy. Guo et al. (2022a,b) assessed the protective effect of a trivalent inactivated vaccine against wild-type *A. paragallinarum* isolates from China. The results indicated that compared to a single vaccination, a two-dose regimen significantly mitigated clinical symptoms, pathological lesions, and bacterial shedding following challenge infection. Notably, in trials targeting the C-type isolate, chickens receiving two doses exhibited no clinical symptom or pathological abnormality post-challenge. Furthermore, no significant difference in body weight or egg production were observed between the two-dose group and the negative control group, demonstrating the safety and efficacy of the vaccine. The study further emphasized that integrating improved ventilation and biosecurity measures is crucial for controlling infectious rhinitis. Although existing trivalent vaccines fail to completely prevent infection by all three serotypes, the findings provide important guidance for clinical vaccine application. Specifically, the research suggests employing a two-dose vaccination strategy in practical settings to enhance vaccine efficacy.

Vaccine efficacy is highly dependent on the representativeness of selected strains and their serotype matching. A study by Melanie et al. revealed significant variations in pathogenicity among strains of different serotypes (A, B, C, and Bvar), with strains within the same serotype exhibiting differing capacities to induce clinical symptoms, horizontal transmission, and septicemic mortality. For example, within serotype A, strains Q2 and Q7 exhibited high virulence, whereas strains Q4 and Q6 caused virtually no symptoms. That finding underscores that vaccine strain selection must not rely solely on serotype classification, but must be grounded in detailed pathogenicity assessments of locally prevalent strains. Huberman’s study specifically highlighted that Bvar strains circulating in Peru possess significant pathogenicity, necessitating future multivalent vaccines to include strains representative of local epidemiological characteristics (Caballero-Garcia et al., 2022). Huberman’s research further underscores the importance of serotype matching. Their study found that both commercial vaccines (V1 and V2) reduced Bvar colonization and clinical signs. The V2 vaccine, containing Bvar strains, demonstrated superior protection effect while the V1 vaccine, lacking this variant, exhibited some cross-protective effect. The study also compared vaccination timing, concluding that the traditional schedule (at 8 and 12 weeks of age) was superior to early vaccination (at 5 and 12 weeks) in reducing clinical signs. However, it recommended considering early vaccination as a strategy on high-risk farms and exploring multi-dose immunization to enhance efficacy (Huberman et al., 2025).

From an immunological mechanism perspective, Robin et al. systematically evaluated the immunostimulatory effects of an octavalent inactivated vaccine targeting avian infectious bronchitis virus (IBV), Newcastle disease virus (NDV), egg-drop syndrome virus (EDSV), and five serotypes of *A. paragallinarum* on the chicken macrophage cell line, HD11. This vaccine effectively induced macrophage release of nitric oxide (NO) and upregulated the expression of pro-inflammatory factors (such as IL-1β and TNF), anti-inflammatory factor IL-10, and Th1/Th17-inducing factor IL-12p40 (van den Biggelaar et al., 2020). Mechanistic studies indicated that the immunostimulatory activity of the vaccine primarily stems from its *A. paragallinarum* antigen, particularly its cell wall component lipopolysaccharide (LPS). Comparative experiments with a trivalent vaccine lacking bacterial antigens and LPS blockade using the antibiotic polymyxin B confirmed the pivotal role of LPS in driving macrophage activation. These findings hold significant clinical implications. Macrophage activation, nitric oxide (NO) production, and the initiation of cytokine networks constitute critical steps in establishing effective immune responses and eliminating pathogens. The study further identified avian paratyphoid antigen, particularly its LPS, as the key component conferring adjuvant-like properties to the vaccine. This suggests that retaining or optimizing such bacterial constituents during vaccine development may enhance overall immunogenicity, especially in inducing early, nonspecific immune defenses. However, the study also highlighted the cytotoxicity of high-concentration vaccines, providing crucial safety reference points for clinical dosage determination.

Compared to inactivated vaccines, attenuated live vaccines induce a more comprehensive immune response point for clinical dosage, thereby reducing the virulence of the pathogen to allow limited replication within the host (Vezina et al., 2021). Guo et al. constructed a transposon-induced random mutation library to screen for a *ksgA* gene-mutated *A. paragallinarum* attenuated strain, designated 2019/HB64-40. Challenge trials demonstrated that infected chickens in the mutant strain group exhibited significantly lower incidences, reduced severity of clinical symptoms, diminished nasal bacterial shedding, and less pathological damage in tissues such as the infraorbital sinus and trachea compared to the wild-type challenge group. Crucially, immunization with this mutant strain as a candidate attenuated live vaccine provided effective protection against challenge with the virulent wild-type strain. Immunized chickens exhibited normal weight gain post-challenge, mild clinical symptoms with rapid recovery, and effectively controlled respiratory bacterial shedding. Histopathological analysis further confirmed that vaccination significantly mitigated lesions, including infraorbital sinus edema, hemorrhage, inflammatory cell infiltration, and tracheal ciliary loss. The findings indicated that the *ksgA* mutant strain 2019/HB64-40 not only exhibited reduced virulence, but also possessed favorable immunogenicity, capable of inducing effective immune protection (Guo M. et al., 2025; Guo Y. et al., 2025).

However, live attenuated vaccines face challenges regarding safety and stability, such as the potential for mild infections in vaccine recipients, and the susceptibility of vaccine strains to reversion mutations or titer decline during passage or storage. Furthermore, in clinical applications, the serotype match of the vaccine directly influences the protective efficacy. Given the multiple serotypes of *A. paragallinarum*, serotype mismatch may result in vaccine failure. Consequently, the serotype coverage of the vaccine and its match with locally circulating strains are critical factors affecting the effectiveness of both traditional inactivated vaccines and attenuated live vaccines.

### 3.2 Novel vaccine technologies

The primary protective antigens of this bacterium such as outer membrane proteins and fimbriae proteins can be expressed and purified *in vitro* (Sun et al., 2026). In recent years, genetic engineering vaccine technology has presented new opportunities for the development of vaccines against *A. paragallinarum*. Such vaccines feature well-defined components, high safety profiles, and the capacity to induce specific immunity against key antigens.

DNA vaccines represent another promising strategy, achieving endogenous antigen expression by directly introducing gene sequences encoding antigens into host cells, thereby simultaneously activating humoral and cellular immune responses. This technology provides advantages of design flexibility and rapid production; however, it typically exhibits weak immunogenicity and requires adjuvants or efficient delivery systems to enhance its immunogenic effect.

To enhance the intensity and stability of vaccine-induced immune responses, the application of novel adjuvants and delivery systems is gaining attention. For example, utilizing bacterial outer membrane vesicles (OMVs) as vaccine carriers not only enables the transport of multiple natural antigens, but also possesses inherent adjuvant activity, significantly amplifying immune responses and potentially offering broader cross-protection (Mei et al., 2020a,b). Furthermore, nanoparticle delivery systems improve antigen stability and delivery efficiency, thereby promoting antigen presentation and immune activation (Luna-Castrejón et al., 2021).

Overall, these novel vaccine technologies demonstrate significant potential to enhance safety, immunological persistence, and broad-spectrum protective capabilities through diversified combinations, providing prospects to address the shortcomings of traditional vaccines. Currently, the development of novel vaccines targeting *A. paragallinarum* remains in the early exploratory phase, yet their theoretical advantages are considerable, presenting broad prospects for advancement.

### 3.3 Challenges and prospects in vaccine application

*A. paragallinarum* exhibit high antigenic diversity and continuous mutation, coupled with frequent mixed infections in clinical settings. These factors collectively increase the complexity of vaccine development and application. Co-infections may not only exacerbate clinical symptoms, but also influence vaccine efficacy through interactions between different pathogens (Guo M. et al., 2025; Guo Y. et al., 2025). Consequently, developing multivalent combination vaccines targeting co-infections represents a crucial future research direction. For example, combining *A. paragallinarum* antigens with those of other co-infecting pathogens to construct multi-pathogen combination vaccines holds promise for achieving synergistic prevention and control against multiple pathogens.

Furthermore, future vaccine development should place greater emphasis on understanding immunological mechanisms. By optimizing immunization protocols and strategies, such as rationally designing vaccination intervals and employing adjuvants, vaccine immunogenicity and durability can be enhanced. Concurrently, leveraging genomics and reverse vaccinology techniques enables systematic screening for highly conserved and immunogenic antigen targets, thereby advancing the development of novel subunit and nucleic acid vaccines. In summary, integrating multidisciplinary approaches to develop combined vaccines targeting multiple pathogens and antigens represents a key pathway for effectively controlling *A. paragallinarum* and its mixed infections.

## 4 Research on drug control and mixed infections of *A. paragallinarum* in poultry

### 4.1 Current status of pharmacotherapy and antimicrobial resistance

*A. paragallinarum* is the primary pathogen responsible for infectious rhinitis in chickens, with antibiotic treatment currently serving as the principal means of controlling outbreaks and transmission. Commonly employed clinical agents include ampicillin, cephalosporins, and fluoroquinolones. However, the widespread use of antimicrobial drugs has led to increasingly severe antibiotic resistance in *A. paragallinarum*, significantly compromising clinical efficacy. Multiple studies indicate that *A. paragallinarum* demonstrates varying degrees of resistance to numerous antibiotics, exhibiting particularly high resistance rates to ampicillin, streptomycin, sulfamethoxazole, and tetracycline. Some strains even display multidrug resistance (Cao et al., 2024). Resistance mechanisms primarily involve the production of β-lactamases, enzymes that hydrolyze β-lactam antibiotics. Secondary antibiotic resistance mechanisms include target site mutations, such as DNA gyrase gene variations that render fluoroquinolones ineffective. Furthermore, the activation of efflux pumps enables bacteria to efficiently expel drugs, thereby enhancing resistance. Monitoring antimicrobial resistance dynamics is crucial for rational drug use. Standardized microdilution methods have been established for *A. paragallinarum* susceptibility testing, providing accurate guidance for clinical prescribing. Consequently, establishing and maintaining resistance surveillance programs is paramount. Microdilution is now the standard method for *A. paragallinarum* susceptibility testing, providing reliable evidence for clinical decision-making (Gütgemann et al., 2024). Furthermore, research has identified multiple resistance genes within the *A. paragallinarum* genome, such as aph(6)-Id, blaTEM-1B, and tet(B), which disseminate resistance through horizontal gene transfer (Mei et al., 2020a,b). Prudent antibiotic use, avoidance of misuse, and the development of novel antimicrobial agents and vaccines represent key strategies for controlling *A. paragallinarum* infections and the spread of resistance (Luna-Castrejón et al., 2021). However, the lack of complete correspondence between resistance phenotypes and genotypes further complicates clinical antimicrobial stewardship. For example, Guo et al. confirmed severe multidrug resistance in *A. paragallinarum* isolates from Hubei Province, where resistance phenotypes did not fully align with genotypes, posing significant challenges for clinical decision-making. Antibiotic susceptibility testing revealed that all strains exhibited resistance to tetracycline and lincomycin, sensitivity to levofloxacin, but varying resistance patterns to multiple other antibiotics, with 58.33% of strains being multidrug resistant. Although resistance gene predictions detected relevant genes for peptidoglycan synthesis, fluoroquinolone, and tetracycline classes across all strains, the highly consistent gene profiles failed to explain the observed phenotypic variations. The disconnect between genes being present but the phenotype absent, or the phenotype being present but the genes not predicted, indicates that gene prediction alone is insufficient to guide clinical decision making; antimicrobial susceptibility test results must be integrated. Furthermore, these unexpressed resistance genes may constitute a genetic reservoir capable of transfer under specific conditions, thereby amplifying the risk of resistance transmission (Guo M. et al., 2025; Guo Y. et al., 2025).

**Figure 4:**
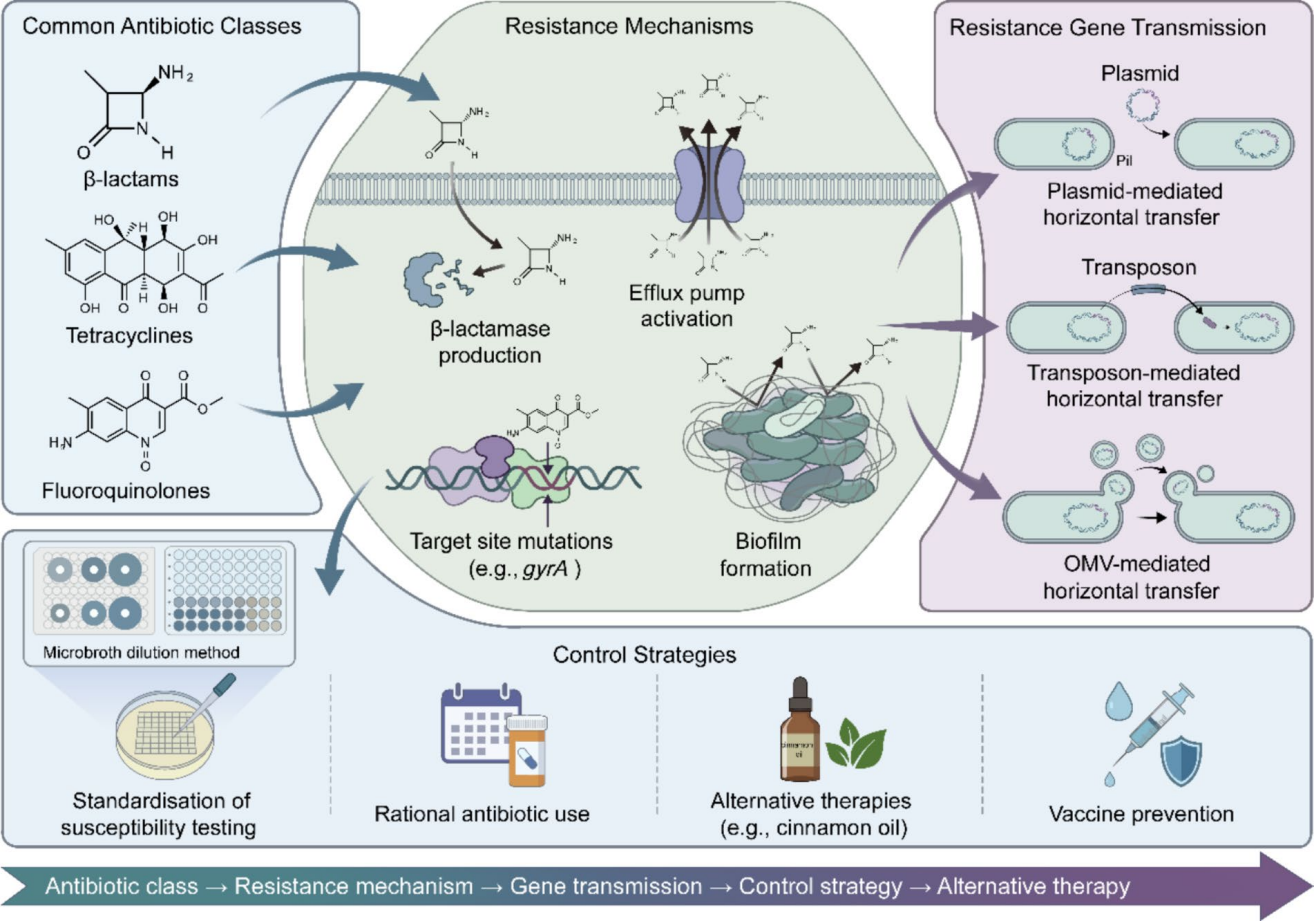
Antibiotic resistance mechanisms of *A. paragallinarum* and control strategies.

To address the challenge of antimicrobial resistance, a comprehensive strategy is urgently required. However, strict adherence to guidelines for the prudent use of antibiotics is essential to prevent misuse, while on the other hand, the active development of novel antimicrobial agents and highly effective vaccines is imperative. Natural products demonstrate potential as alternatives or supplements to antibiotics. For example, natural compounds such as cinnamon oil exhibit antibacterial activity, inhibiting the growth and expression of virulence genes in *A. paragallinarum*, thereby showing promise as antibiotic substitutes. At the phenotypic level, cinnamon oil exhibits significant growth inhibition against multiple pathogens. Using agar disk diffusion and microdilution methods, cinnamon oil effectively suppressed the growth of *S. aureus*, *E. coli*, *Pasteurella multocida and A. paragallinarum*, with corresponding minimum inhibitory concentrations (MICs) determined. However, cinnamon oil exhibited only marginal phenotypic inhibition against *Ornithobacterium rhinotracheale* and *Mycoplasma gallisepticum*, failing to completely halt their growth. At the gene expression level, cinnamon oil demonstrated broader and more profound antimicrobial potential. Real-time quantitative PCR results revealed that cinnamon oil significantly downregulated the expression of key virulence genes in all six tested pathogens. Downregulation ranged from 0.15 to 0.85-fold, with the most pronounced effect observed on the enterotoxin D gene of *S. aureus*. This finding indicated that even for *Ornithobacterium rhinotracheale* and *Mycoplasma gallisepticum,* where phenotypic inhibition was less pronounced, cinnamon oil interfered with pathogenicity at the molecular level by suppressing virulence gene expression, and thereby diminishing bacterial pathogenicity (Erfan and Marouf, 2019). In summary, antimicrobial resistance in *A. paragallinarum* presents a severe and complex challenge. Sustainable control of this disease necessitates enhanced multi-regional surveillance of resistance dynamics, optimization of individualized treatment regimens based on antimicrobial susceptibility testing, and sustained advancement in novel antimicrobial strategies and vaccine development (see Figure 4).

### 4.2 Mechanisms of mixed infections between *A. paragallinarum* and other bacteria in poultry

*A. paragallinarum* frequently co-infects with other pathogens such as *E. coli* and *Pasteurella multocida*, forming complex respiratory infections that significantly exacerbate clinical symptoms and pathological damage. Research indicates that co-infection between *A. paragallinarum* and bacteria such as *E. coli* produces synergistic pathogenic effects through multiple mechanisms. *A. paragallinarum* promotes the colonization and growth of other bacteria, partly due to its release of outer membrane vesicles (OMVs) that carry resistance genes and virulence factors, enhancing the survival of bacterial populations and aiding their colonization (Xu et al., 2022). Furthermore, complex signaling and metabolic interactions exist between *A. paragallinarum* and co-infecting bacteria. Certain resident bacteria, such as *Staphylococcus* spp., directly support *A. paragallinarum* survival and proliferation by supplying essential nutritional factors (e.g., NAD^+^) (Wu et al., 2021). Moreover, mixed infections exacerbate host immune dysregulation, inducing stronger inflammatory responses from the host immune system. Immune evasion mechanisms facilitate prolonged pathogen colonization, leading to worsened symptoms and extended disease duration (Liu et al., 2025).

Bacteria communicate via quorum sensing systems, such as secreting auto-inducer molecules, to coordinate virulence factor expression and biofilm formation, thereby enhancing infection stability and antimicrobial resistance (Chiang et al., 2013). These mechanisms provide a theoretical foundation for developing comprehensive control strategies, including combined antimicrobial therapy, immunomodulation, and interventions targeting bacterial interactions. Research further emphasizes that rationally regulating respiratory microbial community structure to reduce harmful commensal bacteria may help suppress *A. paragallinarum* infection and dissemination (Zhu et al., 2022). Therefore, elucidating the interaction mechanisms between *A. paragallinarum* and co-infecting bacteria is crucial for developing more effective prevention and control strategies.

### 4.3 Research on mixed infections of *A. paragallinarum* and viruses

Mixed infections involving *A. paragallinarum* alongside viruses such as avian influenza virus (AIV) and newcastle disease virus (NDV) are clinically common, exhibiting significant synergistic pathogenic effects that increase mortality and transmission risks. Viral infections typically disrupt the respiratory epithelial barrier, weakening local defense mechanisms and creating favorable conditions for *A. paragallinarum* invasion (Liu et al., 2025). Furthermore, within viral co-infection environments, the biofilm-forming capacity of *A. paragallinarum* may be further enhanced, thereby increasing its drug resistance and pathogenic potential.

At the epidemiological level, the complexity and significance of such mixed infections have been confirmed in multiple field surveillance studies. For instance, Rahman et al. (2021) assessed the prevalence of respiratory diseases and pathogen distribution in small-scale commercial layer farms in Bangladesh using a combination of active surveillance and passive investigations. Between 2017 and 2018, 422 small-scale layer farms in Bangladesh and 22 additional farms were surveyed for six respiratory pathogens using qPCR and conventional PCR. At least one pathogen was detected in 80.91% of farms, with AIV and ILTV being most prevalent. Among AIV-positive farms, H5, H9, and H5/H9 co-detection accounted for 49.12, 15.79, and 22.81%, respectively. Longitudinal monitoring showed that H5/H9 infection caused low mortality (1.05–5.50%) but markedly reduced egg production, with laying rates declining to 12–40% and not returning to baseline after recovery. These findings suggest that the H5 and H9 subtype AIVs are locally endemic, with under-reporting of low pathogenic H5 infections highlighting limitations in current surveillance systems which focus solely on high mortality. The study recommended reducing public health risks through comprehensive vaccination, enhanced biosecurity, and active surveillance incorporating molecular detection.

Another study conducted by Stępień-Pyśniak et al. (2024) reported similar findings, with a high prevalence of respiratory disease and its complex etiological composition following 2 years of monitoring small commercial layer farms in Bangladesh. Among 110 farms experiencing respiratory issues, 80.91% tested positive for one or more respiratory pathogen. AIV and ILTV exhibited the highest detection rates, identified in 57 and 54 farms, respectively. Further typing of AIV-positive samples revealed co-circulation of H5 and H9 subtypes: 28 farms had a single H5 subtype, nine a single H9 subtype, and 13 farms simultaneously hosted both H5 and H9 subtypes, indicating complex co-circulation patterns. Furthermore, co-infection with multiple pathogens was prevalent. Among the 89 positive farms, 51 exhibited mixed infections involving two to four pathogens, 22 farms with AIV and ILTV co-occurring most frequently. Longitudinal monitoring over 20 weeks at 20 infected farms revealed that clinical mortality from H5 or H9 subtype avian influenza virus infections with or without concomitant respiratory pathogens was relatively low. Prior to infection, farm egg production ranged between 80 and 92%. During peak infection, rates plummeted to 12% farms revea productioninical mortality from H5 or H9 subtype avian influenza virus infections, 51 exhibited 72–87%, failing to return to pre-infection levels. The decline in egg production persisted for 26 to 46 days, with no significant difference in the extent of decline between H5 and H9 subtype infections. These data indicate that both highly pathogenic and low pathogenic AIV caused significant economic losses, and that mortality was not the sole indicator for assessing their harmfulness (Paudel et al., 2017).

**Figure 5:**
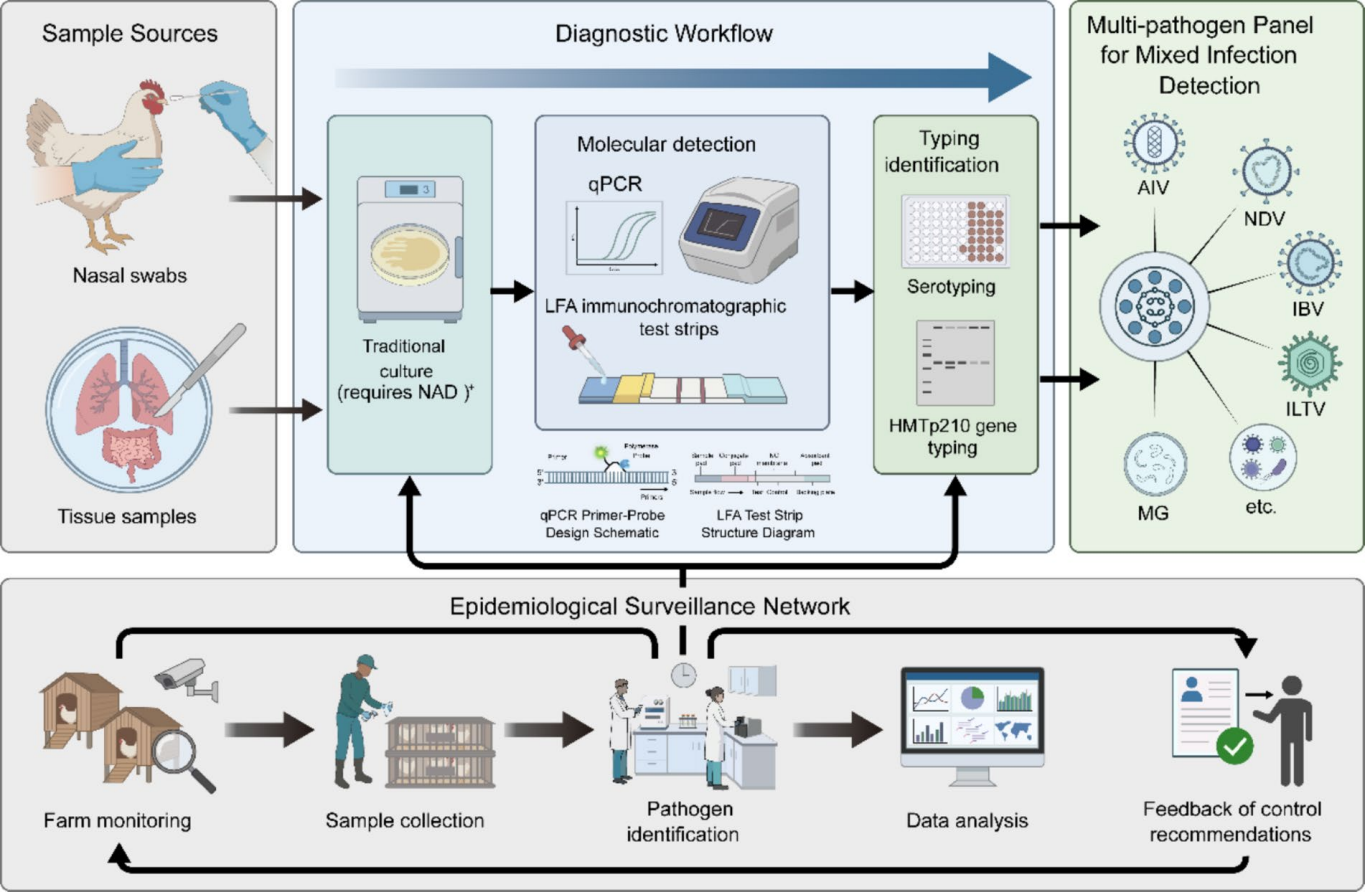
Diagnostic techniques and epidemiological surveillance processes for avian diseases.

The aforementioned research provided crucial field evidence for understanding the pathogenic mechanisms underlying mixed viral and bacterial infections, and described the development of comprehensive control strategies (Li et al., 2026). Such approaches include the development of combined vaccines, the implementation of immunomodulation, and the use of antimicrobial treatments based on precise diagnostics. In summary, mixed infections involving *A. paragallinarum* and viruses involve the interplay of multiple mechanisms, including epithelial barriers, immune regulation, and inflammatory signaling. Deepening our understanding of these interactions will provide key theoretical support for clinical prevention, treatment, and disease control (see Figure 5).

## 5 Conclusion

As the primary pathogen responsible for respiratory diseases such as infectious coryza in chickens, *A. paragallinarum* exhibits significant antigenic diversity and variability, substantially increasing the complexity and difficulty of vaccine development. These characteristics reflect the capacity of the pathogen for environmental adaptation and evasion of immune surveillance, while also indicating that traditional vaccines based on single or limited antigens may have diminished protective efficacy over extended periods of control. Consequently, effectively covering its diverse antigens in next-generation vaccine design has become a critical direction for current and future research.

With advances in genetic engineering and synthetic biology technologies, novel genetically engineered vaccines such as subunit vaccines, nucleic acid vaccines, and broad-spectrum multivalent vaccines provide new possibilities for controlling *A. paragallinarum* infections. By targeting multiple conserved antigens or fusing representative antigens from different serotypes, these vaccines theoretically enable broader and more durable immune protection. Nevertheless, their actual immunogenicity, biosafety, and protective efficacy in complex field environments require thorough validation and optimization through systematic, standardized research. Furthermore, current studies exhibit discrepancies in vaccine efficacy assessment criteria, challenge strain selection, and immunization protocols. This underscores the necessity for the comprehensive evaluation of multiple lines of evidence when formulating control strategies, striving for objectivity and thoroughness.

In pharmacotherapy, antibiotics serve as a crucial tool for controlling acute outbreaks, yet their efficacy faces increasingly severe challenges from antimicrobial resistance. The inappropriate use of antimicrobial agents not only leads to treatment failure, but also accelerates the selection and spread of multidrug-resistant strains. Professional countermeasures must encompass strict adherence to precision prescribing based on antimicrobial susceptibility testing, establishing continuous resistance surveillance networks, and encouraging the development of novel antimicrobial agents or alternative products, such as antimicrobial peptides and bacteriophages. Furthermore, exploring combination therapies that integrate immunomodulators with antimicrobial treatments has promise for enhancing clearance efficiency by improving the immune status of the host. Overall, therapeutic strategies should be closely coordinated with preventive measures such as biosecurity protocols and vaccination programs to establish a multi-tiered, comprehensive control system.

Moreover, *A. paragallinarum* frequently co-infects with other bacteria and viruses in clinical settings. This co-infection mechanism further exacerbates disease severity and complicates control efforts. Mixed infections not only influence pathogen virulence and immune evasion, but may also trigger abnormal immune activation or suppression, increasing treatment complexity. Current understanding of the molecular mechanisms underlying mixed infections remains incomplete, with research predominantly focused on single pathogens and lacking systematic analysis of interactions. There is an urgent need to strengthen systematic investigations into pathogen-host interactions during co-infection, elucidating how these interactions influence disease progression. Such investigations will provide a scientific basis for developing comprehensive prevention and control strategies.

The prevention and control of respiratory diseases associated with *A. paragallinarum* constitutes a systematic endeavor, confronting multiple challenges, including antigenic variation, drug resistance, and mixed infections. This necessitates interdisciplinary convergence and collaborative innovation. Research priorities should focus on elucidating antigenic drift patterns and immune evasion mechanisms to guide the development of broader-spectrum, safer multivalent or universal vaccines. Concurrently, deepening understanding of the ecology and molecular mechanisms of mixed infections is essential to refine risk-based integrated management strategies.

On the therapeutic front, adherence to prudent antimicrobial stewardship principles must be upheld, alongside proactive advancement of novel therapeutic agents. Ultimately, only through an integrated strategy combining precision vaccine immunization, scientifically-informed drug therapy, and enhanced biosecurity management can *A. paragallinarum* be effectively controlled, safeguarding the health and stable production of the poultry farming industry.
